# HIF-1-alpha links mitochondrial perturbation to the dynamic acquisition of breast cancer tumorigenicity

**DOI:** 10.18632/oncotarget.8570

**Published:** 2016-04-04

**Authors:** Ching-Ying Kuo, Chun-Ting Cheng, Peifeng Hou, Yi-Pei Lin, Huimin Ma, Yiyin Chung, Kevin Chi, Yuan Chen, Wei Li, Hsing-Jien Kung, David K. Ann

**Affiliations:** ^1^ Diabetes and Metabolism Research Institute, City of Hope, Duarte, CA, USA; ^2^ Irell & Manella Graduate School of Biological Sciences, City of Hope, Duarte, CA, USA; ^3^ Department of Oncology, Fujian Medical University Union Hospital, Fuzhou, Fujian, China; ^4^ Fujian Key Laboratory of Translational Cancer Medicine, Fuzhou, Fujian, China; ^5^ Integrated Laboratory, Center of Translational Medicine, Taipei Medical University, Taipei, Taiwan, ROC; ^6^ Department of Molecular Medicine, Beckman Research Institute, City of Hope, Duarte, CA, USA; ^7^ Department of Dermatology, Keck School of Medicine, University of Southern California, Los Angeles, CA, USA; ^8^ Institute of Molecular and Genomic Medicine, National Health Research Institutes, Taiwan, ROC

**Keywords:** α-ketoglutarate, mitochondria, metabolic reprogramming, hypoxia-inducible factor-1α (HIF-1α), breast cancer tumorigenicity

## Abstract

Up-regulation of hypoxia-inducible factor-1α (HIF-1α), even in normoxia, is a common feature of solid malignancies. However, the mechanisms of increased HIF-1α abundance, and its role in regulating breast cancer plasticity are not fully understood. We have previously demonstrated that dimethyl-2-ketoglutarate (DKG), a widely used cell membrane-permeable α-ketoglutarate (α-KG) analogue, transiently stabilizes HIF-1α by inhibiting prolyl hydroxylase 2. Here, we report that breast cancer tumorigenicity can be acquired through prolonged treatment with DKG. Our results indicate that, in response to prolonged DKG treatment, mitochondrial respiration becomes uncoupled, leading to the accumulation of succinate and fumarate in breast cancer cells. Further, we found that an early increase in the oxygen flux rate was accompanied by a delayed enhancement of glycolysis. Together, our results indicate that these events trigger a dynamic enrichment for cells with pluripotent/stem-like cell markers and tumorsphere-forming capacity. Moreover, DKG-mediated metabolic reprogramming results in HIF-1α induction and reductive carboxylation pathway activation. Both HIF-1α accumulation and the tumor-promoting metabolic state are required for DKG-promoted tumor repopulation capacity *in vivo*. Our data suggest that mitochondrial adaptation to DKG elevates the ratio of succinate or fumarate to α-KG, which in turn stabilizes HIF-1α and reprograms breast cancer cells into a stem-like state. Therefore, our results demonstrate that metabolic regulation, with succinate and/or fumarate accumulation, governs the dynamic transition of breast cancer tumorigenic states and we suggest that HIF-1α is indispensable for breast cancer tumorigenicity.

## INTRODUCTION

Altered energy metabolism, a hallmark of cancer, plays a key role in tumor development for rapid proliferation, maintenance of redox homeostasis, and epigenetic reprogramming [1, 2]. Experimental evidence has supported the hypothesis that various metabolic enzymes are reprogrammed by oncogenes and dysfunctional or non-functional tumor suppressor genes [3, 4]. Consequently, the metabolic profiles in proliferating cancer cells are shifted toward glycolysis, even though there is sufficient oxygen available to support oxidative phosphorylation (OXPHOS); this shift to glycolysis is also known as the Warburg effect [5, 6]. Recently, attention has turned to the mitochondria and their role in promoting stem cell self-renewal and tumorigenic cell expansion [7–10]. Implicit in the ability of cancer cells to switch metabolic states is the concept that alternating between differing metabolic states is required to effectively adapt to the rapidly changing tumor nutritional microenvironment.

There is increasing awareness that individual breast cancer (BC) tumors contain a heterogeneous cell population, and that this intratumoral heterogeneity contributes to therapy failure and disease progression [1]. In the past decade, studies have largely focused on the unidirectional hierarchy from tumorigenic cells, so-called cancer stem cells (CSCs) to non-tumorigenic progeny, emphasizing the critical role of CSCs in cancer development, treatment resistance and metastasis [11]. Recently, however, it has been proposed that non-stem-like cancer cells can spontaneously acquire or preserve CSC-like properties [12–14]. Although emerging studies reveal that CSCs are metabolically distinct from cancer cells, no studies have explored the tumor nutritional microenvironment and its effects on the dynamics of CSC-like subpopulations.

Hypoxia-inducible factor-1α (HIF-1α) is a master transcriptional regulator that responds to a hypoxic environment and induces the transcription of genes, including vascular endothelial growth factor (*VEGF*), glucose transporter 1 (*GLUT1*) and pyruvate dehydrogenase kinase 1 (*PDK1*), which promote vascularization and tumor cell survival [15]. For example, overexpression of HIF-1α is associated with BC metastasis and poor survival of BC patients [16–19]. In some cancers, accumulation of HIF-1α can result from loss-of-function mutations in proteins that cause HIF-1α degradation, such as the Von Hippel-Lindau tumor suppressor (pVHL) or the enzymes that produce cofactors for the prolyl hydroxylases (PHDs). For example, sporadic renal cell carcinomas with mutations or deletions in the *VHL* gene exhibit elevated HIF-1α levels [20]. In addition, mutations in succinate dehydrogenase (SDH) and fumarate hydratase (FH), enzymes that produce competitive metabolites for PHD cofactors, are found in cancers [21–30]. SDH and FH hydrolyze succinate and fumarate, respectively, to fuel the tricarboxylic acid (TCA) cycle. Mutations in SDH or FH cause succinate or fumarate to accumulate and compete with α-ketoglutarate (α-KG) for PHD binding, thereby inhibiting PHD and stabilizing HIF-1α [31, 32]. Mutations have also been identified in isocitrate dehydrogenase 1 (IDH1) that inhibit IDH1 catalytic activity in gliomas, thereby reducing the production of α-KG, inhibiting PHD, increasing HIF-1α, and presumably, promoting tumorigenesis [33].

Although the mechanism is not totally understood, some evidence suggests that α-KG can increase the stem or stem-like potential of embryonic stem cells (ESCs) [34]. Here, we have addressed this fundamental biological question in the context of BC cell metabolic state. Our laboratory initially identified that dimethyl-2-ketoglutarate (DKG), which has been widely used as an α-KG-supplement [35, 36], transiently stabilizes HIF-1α by inhibiting PHD2-mediated hydroxylation/degradation of HIF-1α under normoxia [37]. HIF-1α, along with its complex signaling network, has been proposed as a key mediator of BC malignancies [16, 38]. Nonetheless, nothing is known about the mechanism of DKG-induced PHD2 inhibition and the consequences of prolonged DKG exposure on BC cells. Here, we studied the CSC-like properties of a panel of established and patient-derived BC cells treated with DKG. The metabolic and transcriptional landscape and the underlying mechanism were analyzed. We found that sustained DKG treatment triggered the accumulation of succinate and fumarate, while reducing the abundance of mRNAs encoding SDH, FH, and subunits of the mitochondrial electron transport chain (ETC) complex I and V. Our data suggest that differential regulation of mitochondrial respiration, glycolysis and fatty acid oxidation (FAO), coupled with accumulated HIF-1α, aggravate tumorigenicity *in vivo*. To our knowledge, this is the first use of a metabolite precursor, DKG, to perturb mitochondrial function, which in turn regulates BC tumorigenicity. Together, our results provide insight into how the tumor metabolic state is dynamically regulated to promote breast cancer aggressiveness.

## RESULTS

### DKG causes HIF-1α induction, succinate and fumarate accumulation, and transcriptional reprogramming in BC cells

**Figure 1:**
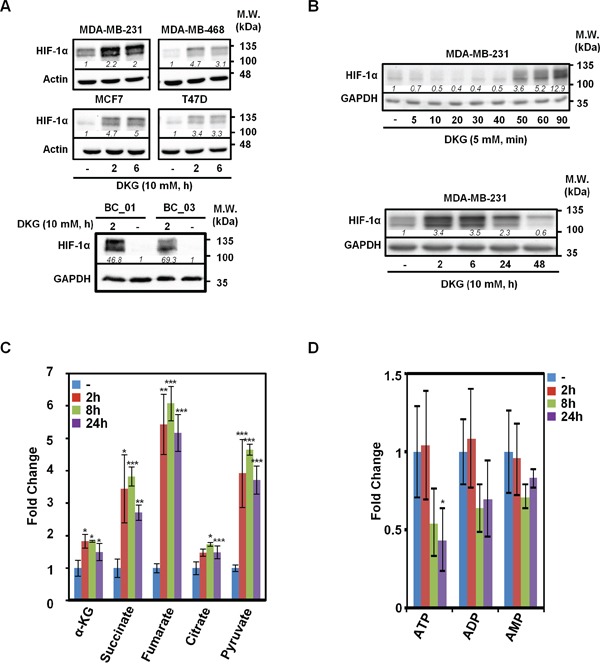
Dimethyl-2-ketoglutarate (DKG) induces HIF-1α and alters the metabolic profile in breast cancer (BC) cells **A.** DKG stabilizes HIF-1α in multiple BC cells. HIF-1α levels in the DKG-treated BC cells (10 mM, 2 and 6 h) were assessed by western blot analysis. **B.** The induction of HIF-1α by DKG is fast and transient. HIF-1α levels in DKG-treated MDA-MB-231 cells were assessed by western blot analysis. **C, D.** Succinate and fumarate accumulate (C) and ATP is decreased (D) in DKG-treated cells. Metabolites in the MDA-MB-231 cells treated with DKG (10 mM, 0, 2, 8, 24 h) were analyzed by NMR spectroscopy. n = 3, _*_: *p* < 0.05; _**_: *p* < 0.01; _***_: p < 0.005. (A, B) One representative blot from n = 3 is shown. *Italic numbers* indicate the relative protein level.

To extend our previous observations [37], multiple BC cell lines were treated with a pharmacological dose of DKG (10 mM). Consistent with our initial results, DKG induced HIF-1α in four established BC cell lines (Figure 1A, *upper panel*) and two primary BC cell lines (Figure 1A, *lower panel*). The induction of HIF-1α was rapid (within 2 h after treatment [Figure 1B, *upper panel*]) and transient (Figure 1B, *lower panel*). In the remaining studies, BC cells were replenished with DKG daily to sustain HIF-1α at a higher level. To understand intracellular action of DKG, we measured several key intermediary metabolites in the TCA cycle using NMR-based metabolomic analysis. The intracellular levels of α-KG, succinate, fumarate, citrate and pyruvate all increased significantly, albeit with different kinetics and to varying degrees, within 24 h after DKG treatment (Figures 1C and S1A). Notably, although we treated BC cells with a pharmacological dose of DKG (10 mM), the intracellular level of α-KG remained in the physiological range (~μM) (Figure S1A), excluding the possibility that the effects we observed were due to excess intracellular α-KG. The disproportionate increase in these metabolites elevated the ratios of succinate to α-KG (from 0.54 to 1.02, 1.14 and 0.99) and fumarate to α-KG (from 0.11 to 0.32, 0.36 and 0.38) in a time-dependent manner (Figure S1B). Because we also observed a time-dependent ATP reduction (Figure 1D), we speculated that the DKG-fueled TCA cycle was uncoupled from ATP generation. Because succinate and fumarate are known to inhibit PHD2 [28, 31, 32], their accumulation in the DKG-treated cells corroborated our previous observations that DKG stabilized HIF-1α through inhibiting PHD2-mediated hydroxylation [37].

Because HIF-1α is known to regulate transcription, we therefore compared the gene expression profiles in MDA-MB-231 cells and two primary BC cells with or without DKG administration by performing RNA-sequencing (RNA-seq) analysis. The top five DKG-affected pathways were HIF-1α signaling, ubiquinol-10 biosynthesis, cell cycle control, chromosomal replication and TGF-β signaling (Figure S1C). We concluded that DKG treatment, in addition to inducing HIF-1α (Figure 1A), creates a pseudohypoxic state under normoxia. From our RNA-seq analysis, we also observed that the message abundance of *SDH* and *FH* was down-regulated in the DKG-treated cells (Figure S1D). We further postulated that the increase in both succinate and fumarate, as well as the decrease in *SDH* and *FH* mRNA levels, resulted in an imbalance of TCA metabolites. This metabolite imbalance could then impair PHD2 activity, thereby stabilizing HIF-1α and reprogramming the transcriptional landscape in BC cells.

### DKG promotes the acquisition of breast cancer stem cell-like properties

HIF-1α signaling has been proposed to be a key mediator of BC malignancies [16, 38]; we therefore investigated the effects of prolonged DKG treatment on the tumorigenic properties of BC cells. Prolonged treatment with DKG (10 days) reduced the clonogenicity of MDA-MB-231 cells (Figure S1E, *upper panel*). However, after terminating DKG treatment, DKG pre-treated cells resumed proliferation and exhibited comparable colony numbers to untreated cells (Figure S1E, *lower panel*), ruling out the hypothesis that DKG is toxic and suggesting that its effect is dynamic and reversible. In parallel, the subset of Ki67^Low^/Hoechst 33342^Low^ cells, representing the G0 quiescent subpopulation, increased in DKG-treated MDA-MB-231 cells (Figure S1F, *upper panel*, *lower-left quadrants*). After 4 to 7 days of DKG treatment, the G0 subpopulation increased by approximately 2-fold (12.7% to 24.5% and 31.3%) (Figure S1F, *lower panel*).

**Figure 2:**
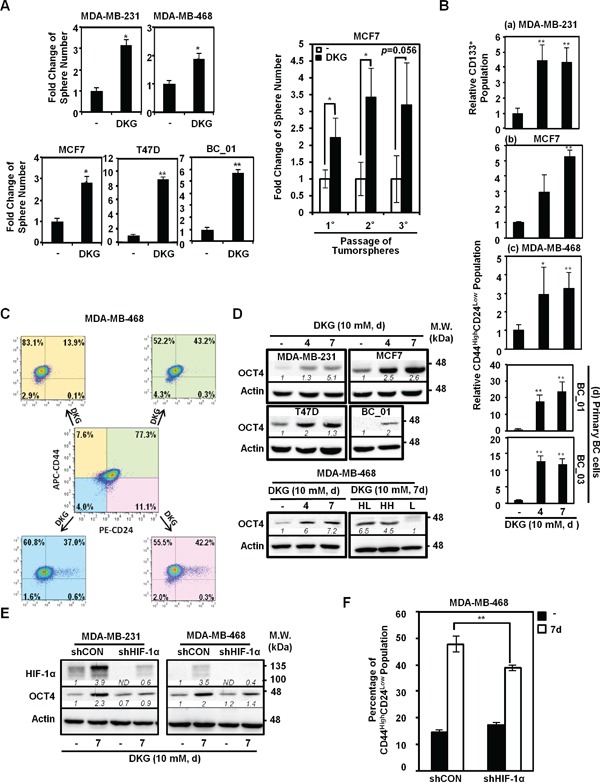
DKG promotes tumorigenic properties in BC cells **A.** DKG pre-treatment promotes tumorsphere formation. *Left panel*: Quantification of tumorspheres formed by the untreated and DKG pre-treated BC cells. *Right panel*: *In vitro* serial passaging of tumorspheres formed by the untreated and DKG-treated MCF7 cells. _*_: *p* < 0.05; _**_: *p* < 0.01, n = 3. **B.** DKG regulates the abundance of cancer stem cell (CSC) surface markers in BC cells. Flow cytometric analyses of surface markers in DKG-treated BC cells (10 mM, 4, 7 days). CD133 was assessed in MDA-MB-231 cells (a). CD44 and CD24 were assessed in MCF7 (b), MDA-MB-468 (c) and primary BC cells (d). The percentage of CD133-positive or CD44^High^CD24^Low^ subpopulations in the untreated sample was set as 1. Bar graphs represent the mean ± SD, n = 3. **C.** DKG converts non-tumorigenic subpopulations to tumorigenic subpopulations. MDA-MB-468 cells were sorted based on CD44 and CD24 expression. Sorted cells were treated with DKG (10 mM, 7 days). CD44 and CD24 expression was assessed. APC: allophycocyanin-conjugated. PE: phycoerythin-conjugated. Representative graphs. n = 3. **D.** DKG elevates OCT4 abundance. *Upper panel*: OCT4 levels in multiple BC cell lines treated with DKG (10 mM for 4 or 7 days) were determined by western blot analysis. *Lower panel*: OCT4 is expressed in the CD44^High^ subpopulations after DKG treatment. The DKG-treated MDA-MB-468 cells (10 mM, 7 days) were sorted into CD44^High^CD24^Low^ (HL), CD44^High^CD24^High^ (HH) and CD44^Low^ (L) subpopulations for analyzing OCT4 expression by western blot analysis. Representative blots. n = 3. **E.** DKG-induced OCT4 expression depends on HIF-1α. **F.** HIF-1α is responsible for inducing a tumorigenic subpopulation in BC cells. _**_: *p* < 0.01, n = 3. (D, E) One representative blot from n = 3 is shown. *Italic numbers* indicate the relative protein level. ND: not detectable.

Given that tumorigenic cells can grow as 3-dimensional non-adherent structures when clonally seeded in serum-free media, we pre-treated a panel of established and primary BC cells with DKG (10 mM) for 7 days and subjected the cells to tumorsphere formation assays. Consistently, DKG pre-treatment increased both the size (Figure S2A) and the number (Figure 2A, *left panel*) of tumorspheres in multiple BC cells. In addition, DKG pre-treatment supported the *in vitro* propagation of tumorspheres (Figure 2A, *right panel*), suggesting that DKG enriches the stem-like subpopulation in culture.

To further characterize the tumorsphere-promoting effects of DKG, flow cytometric analyses were performed to examine the abundance of CD44, CD24 and CD133. These are cell surface markers frequently used to identify CSCs in BC [39–42]. Because more than 70% of MDA-MB-231 cells exhibit the CD44^High^CD24^Low^ phenotype [39] and CD133 expression is highly correlated with the invasiveness and poor prognosis of triple-negative BC (TNBC) [40, 42], we assessed whether DKG treatment increased the surface expression of CD133 in MDA-MB-231 cells by flow cytometry. A 4-fold increase in the percentage of CD133-positive was observed in DKG-treated MDA-MB-231 cells (4 and 7 days; Figures 2B, *panel a*, and S2B). In parallel, the CD44^High^CD24^Low^ subpopulation, within the bulk of MCF7 cells, was elevated from 1.8% to 6.4% and 9.5% following 4 and 7 days of DKG treatment (Figures 2B, *panel b*, and S2C). Likewise, the CD44^High^CD24^Low^ subpopulation also increased in the DKG-treated MDA-MB-468 cells, by approximately 3-fold (Figures 2B, *panel c*, and S2D). In addition, DKG treatment caused a significant enrichment of the CD44^High^CD24^Low^ subpopulation in two patient-derived primary BC cell lines, BC_01 and BC_03 (Figures 2B, *panel d*, and S2E). To differentiate whether DKG-mediated modulation in cell surface marker expression was stable or transient, we performed fluorescence-activated cell sorting (FACS) to enrich for four subpopulations, according to the surface expression of CD44 and CD24 in MDA-MB-468 cells, and we then incubated the respective subpopulations with DKG for an additional 7 days. We found that DKG not only expanded the CD44^High^CD24^Low^ subpopulation, but also converted the other three non-CD44^High^CD24^Low^ subpopulations to CD44^High^CD24^Low^ (Figure 2C). These results suggest that DKG treatment enriched for the subpopulation of BC cells with increased tumorsphere formation capacity and increased CSC surface marker abundance. These two traits are frequently used as surrogate markers for tumorigenic CSC-like properties.

Next, we examined the expression of pluripotency factors, which were reported to express at higher levels in breast CSC-like cells and are associated with BC progression [43–47]. For example, the subpopulation of BC cells expressing a high level of octamer-binding transcription factor 4 (OCT4) displays tumorigenic properties [43, 48, 49]. After DKG treatment for 4 and 7 days, the steady-state OCT4 protein abundance was elevated in multiple BC cell lines (Figure 2D, *upper panel*). Notably, OCT4 was enriched in the sorted CD44^High^ subpopulations (Figure 2D, *lower panel*). In parallel, the mRNA abundances of pluripotency factors, including *OCT4*, *NANOG*, *SOX2, MYC* and *KLF4*, as well as the cell surface marker *CD44*, increased in DKG-treated MDA-MB-231 and MCF7 cells (Figure S2F). Altogether, these results demonstrated that DKG induced the expression of surrogate markers for the CSC-like phenotype in multiple established and primary BC cells.

Lastly, we assessed whether the up-regulation of the surrogate markers for the tumorigenic phenotype was dependent on HIF-1α. Knocking down HIF-1α by a short hairpin (sh)RNA reduced the induction of OCT4 and CSC surface markers in DKG-treated cells (Figures 2E and 2F), suggesting the DKG-induced expression of OCT4 and the CSC surface markers was HIF-1α-dependent. Taken together, these data suggest that DKG treatment promotes the accumulation of succinate and fumarate, resulting in HIF-1α induction and changes in subsequent transcriptional reprogramming and thereby impacting multiple cellular processes in BC cells.

### DKG enhances tumorigenic capacity

**Figure 3:**
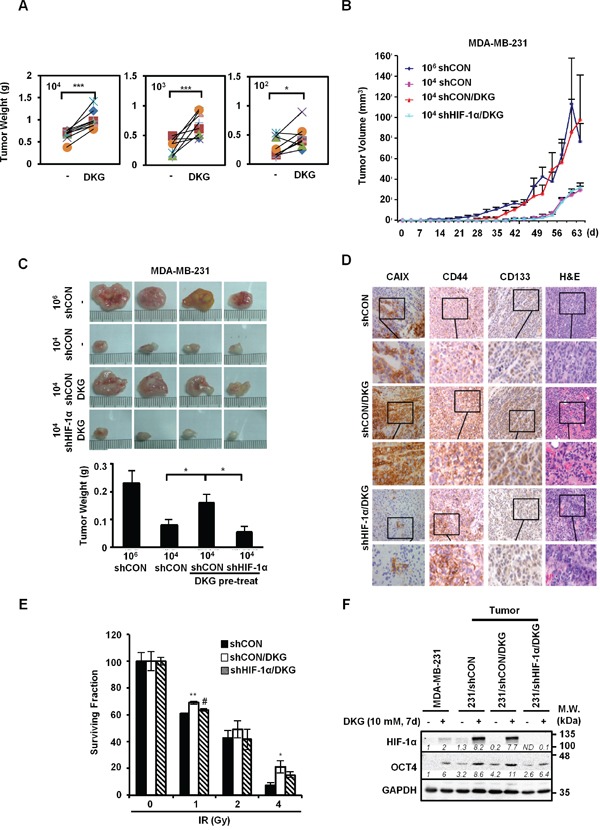
DKG augments the tumorigenic properties of BC cells *in vivo* **A.** DKG enhances the growth of tumors *in vivo*. The untreated or DKG pre-treated MDA-MB-231 cells were injected subcutaneously in the left and right flank of the same NSG mouse in serial dilutions (10^4^, 10^3^, 10^2^ cells). The plot shows the comparison of corresponding tumor weight (g), measured after harvesting on day 60, in the same mouse. _*_: *p* < 0.05; _***_: *p* < 0.005, n = 8. **B, C.** DKG augments the tumorigenic properties of BC cells through HIF-1α *in vivo*. (B) Different numbers of MDA-MB-231/shCON or shHIF-1α cells with or without pre-treatment with DKG were injected into NSG mice. Tumor volume was measured twice weekly for 65 days and tumors were harvested on day 65. (C) Images show that pre-treatment with DKG boosted expansion of xenografted MDA-MB-231 cells in a HIF-1α-dependent manner (*upper panel*). Tumor weight (g) was measured after harvesting on day 65 and is shown as mean ± SD (*lower panel*). _*_: *p* < 0.05. n = 4. **D.** Pre-treatment with DKG increases CAIX abundance in xenografted tumors. Immunohistochemical (IHC) staining of CAIX, CD44, CD133 and H&E staining of the tumors. Bar: 50 μm. Insets show the enlargement of the indicated areas. **E.** DKG confers relative resistance of BC cells to irradiation. DKG-pre-treated xenografted MDA-MB-231/shCON and MDA-MB-231/shHIF-1α cells were subjected to a clonogenic survival assay followed by irradiation. Colonies were counted, corrected for plating efficiency and presented as the surviving fraction relative to untreated controls. _*_: *p* < 0.05; _**_: *p* < 0.01, shCON/DKG compared with shCON; #: *p* < 0.05, shCON/DKG compared with shHIF-1α/DKG. **F.** Harvested tumor cells from xenografted tumors are still responsive to DKG. HIF-1α and OCT4 levels in MDA-MB-231 cells harvested from xenografted tumors were assessed by western blot analysis. One representative blot from n = 3 is shown. *Italic numbers* indicate the relative protein level. ND: not detectable.

Mouse xenografts assay was used to confirm the effects of DKG on promoting the tumorigenic properties of MDA-MB-231 cells *in vivo*. Serial dilutions (10^4^, 10^3^, 10^2^ cells) of untreated and DKG pre-treated MDA-MB-231 cells were injected in the left and right flanks of the same NOD/SCID/IL-2Rγ null (NSG) mouse, respectively. Visible tumors were formed on the both flanks in all groups of mice (n = 8 for each dilution). Consistent with results shown in Figure 2, DKG pre-treated cells gave rise to bigger tumors than the vehicle pre-treated cells (Figure 3A). Next, to determine the contribution of HIF-1α in DKG-mediated tumor propagation, MDA-MB-231/shCON or/shHIF-1α cells (10^4^) were pre-treated with DKG, and then injected into the flanks of NSG mice. For comparison, we also injected 10^6^ vehicle pre-treated MDA-MB-231/shCON cells. Measurable tumors formed in the mice injected with 10^6^ MDA-MB-231/shCON cells by day 30 after engrafting. Strikingly, after 40-days of engraftment, 10^4^ DKG-pretreated MDA-MB-231/shCON cells propagated rapidly to form tumors similar in size to the tumors formed by 10^6^ MDA-MB-231/shCON cells. In contrast, neither 10^4^ untreated MDA-MB-231/shCON cells nor 10^4^ DKG-pretreated MDA-MB-231/shHIF-1α cells formed measurable tumors (Figure 3B). Tumor sizes and weights at day 65 after engraftment are shown in Figure 3C. 10^4^ DKG-pretreated MDA-MB-231/shCON cells produced tumors with sizes comparable to those from 10^6^ untreated MDA-MB-231/shCON cells. Next, we examined a well-established HIF-1α downstream protein, carbonic anhydrase 9 (CAIX), in the xenograft tumors by immunohistochemistry (IHC) and found a significant increase in CAIX signals in the DKG-pretreated tumors (Figure 3D). As expected, the level of CAIX was much lower in the HIF-1α knockdown tumors. This result suggests that DKG increases CAIX abundance via HIF-1α signaling. Consistent with our *in vitro* study, we observed an increase of CD44 and CD133 in DKG-pretreated tumors and HIF-1α knockdown reduced the intensity of CD44 and CD133. Moreover, we also observed more vascularization in DKG-pretreated tumors in a HIF-1α-dependent manner, consistently indicating an increase in HIF-1α signal in the context of DKG treatment (Figure 3D).

Another important breast CSC-like feature is treatment resistance. To address this possibility, we established cell lines from the harvested tumors, treated them with DKG for 7 days, and performed a clonogenic survival assay to evaluate cell survival following ionizing radiation (IR). As shown in Figure 3E, the DKG-treated tumor cells were relatively resistant to IR, and this phenomenon was reversed in the HIF-1α knockdown tumor cells. The observation of a lack of OCT4 induction in DKG-treated cells derived from MDA-MB-231/shHIF-1α-xenografted tumors (Figure 3F) supported the notion that both HIF-1α and DKG-mediated metabolic adaptation are required for the expansion of cells with tumorigenic properties.

### DKG increases oxygen consumption rate

**Figure 4:**
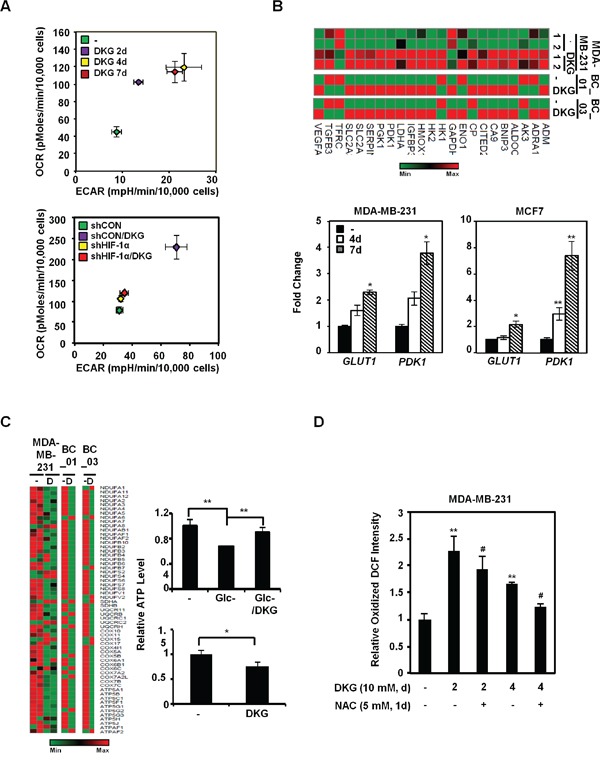
DKG enhances metabolic flux in BC cells **A.** Energy metabolism is enhanced by DKG-induced HIF-1α in MDA-MB-231 cells. The oxygen-consumption rate (OCR) and extracellular acidification rate (ECAR) were measured during the course of DKG treatment in MDA-MB-231 cells *(upper panel*) and in MDA-MB-231/shCON versus MDA-MB-231/shHIF-1α cells (*lower panel*). The PhenoGram profiles show the metabolic switch of OCR and ECAR at the sixth point (22.5 mins after Glc injection during the ECAR measurement) for the ECAR versus OCR plots, as shown in Figures S3A and S3B; n = 3. **B.** DKG increases the message abundance of genes regulating glucose metabolism. *Upper panel*: heat map generated from RNA-seq analysis of the untreated and DKG-treated MDA-MB-231 cells and primary BC cells. *Lower panel*: qRT-PCR analysis revealed that glucose transporter 1 (*GLUT1*) and pyruvate dehydrogenase kinase 1 (*PDK1*) mRNA levels are elevated in DKG-treated MDA-MB-231 cells (10 mM, 4, 7 days). _*_: *p* < 0.05; _**_: *p* < 0.01, n = 3. **C.** Genes regulating the mitochondrial electron transport chain are reduced by DKG treatment. *Left panel*: heat map generated from RNA-seq analysis. *Right panel*: MDA-MB-231 cells were cultured in Glc-depleted medium (upper *right panel*) or Glc-complete (25 mM) medium (*lower right panel*) supplemented with or without DKG for 48 h. The ATP levels were measured using a luciferase assay. _*_: *p* < 0.05; _**_: *p* < 0.01. n = 3. **D.** The levels of reactive oxygen species (ROS) increase in DKG-treated cells. ROS levels were measured by DCFDA staining. The oxidized DCF signal is shown. _**_: *p* < 0.01 (DKG-treated *vs.* untreated); #: *p* < 0.05 (DKG+NAC *vs.* DKG only), n = 3.

To investigate how DKG impacts metabolic pathways, we measured the metabolic flux in the DKG-treated BC cells. Intriguingly, DKG treatment first preferentially increased the oxygen consumption rate (OCR) (day 2), and then mainly elevated the extracellular acidification rate (ECAR) (day 4), with both values reaching a plateau by day 4 (Figures 4A, *upper panel*, and S3A). The enhancement of both OCR and ECAR was diminished by HIF-1α knockdown (Figures 4A, *lower panel*, S3B). Interestingly, this increase in basal OCR was not abrogated by oligomycin treatment (Figures S3C and S3D, *panel a*), suggesting an increase in the oligomycin-insensitive fraction, which could be caused by proton leakage from the intermembrane space [50]. However, additional analysis indicated that the increased proton leakage in DKG-treated cells was very modest (Figure S3D, *panel b*). Instead, a marked expansion of non-mitochondrial respiration was detected in DKG-treated cells (Figure S3D, *panel c*). In addition, an increase in the maximal respiration (uncoupled respiration) was observed in DKG-treated cells (Figure S3D, *panel d*). We therefore concluded that one of the DKG-associated metabolic phenotypes was the elevated respiration in the setting of mitochondrial uncoupling, coexisting with reduced ATP (Figure 1D). Moreover, the increase in uncoupled oxygen utilization was HIF-1α-dependent, as knockdown of HIF-1α significantly dampened the DKG-induced basal, non-mitochondrial and maximal respiration (Figures S3C and S3D, *panels a*, *c*, *d*).

To explore the underlying mechanism(s), we next evaluated the mRNA abundance of glycolytic genes from our RNA-seq data. Consistent with Figure 4A, the message abundance of glycolytic genes was up-regulated in DKG-treated MDA-MB-231 and two primary BC cells (Figure 4B, *upper panel*). The abundance of the *GLUT1* and *PDK1* messages was further validated by qRT-PCR in MDA-MB-231 and MCF7 cells treated with DKG (Figure 4B, *lower panels*). In contrast, relative message abundances for mitochondrial ETC proteins, especially complexes I and V, decreased after DKG treatment (Figure 4C, *left panel*). Indeed, unlike its role in restoring steady-state ATP level in the cells deprived of glucose (Glc) for 48h (Figure 3C, *upper right panel*), but consistent with Figure 1D, prolonged DKG treatment reduced ATP levels in the cells cultured in nutrient-rich conditions (Figure 4C, *lower right panel*). It is also possible that increased α-KG (Figure 1C) binds and inhibits ATP synthase to reduce ATP production [51]. Lastly, given that DKG increased OCR, which could serve as a source of intracellular ROS, we examined the ROS levels in the DKG-treated cells by 2′,7′-dichlorofluorescin diacetate (DCFDA) labeling. After 2 and 4 days of DKG treatment, a modest, albeit significant, N-acetylcysteine (NAC)-sensitive increase in oxidized 2′,7′-dichlorofluorescin (DCF) signal was observed (Figure 4D). Taken together, our data support a model where DKG regulates specific sets of genes to increase both OCR and ECAR in BC cells, while uncoupling the ETC with ATP synthesis, preventing a massive surge in ROS.

### DKG increases reductive carboxylation and FAO beyond HIF-1α

**Figure 5:**
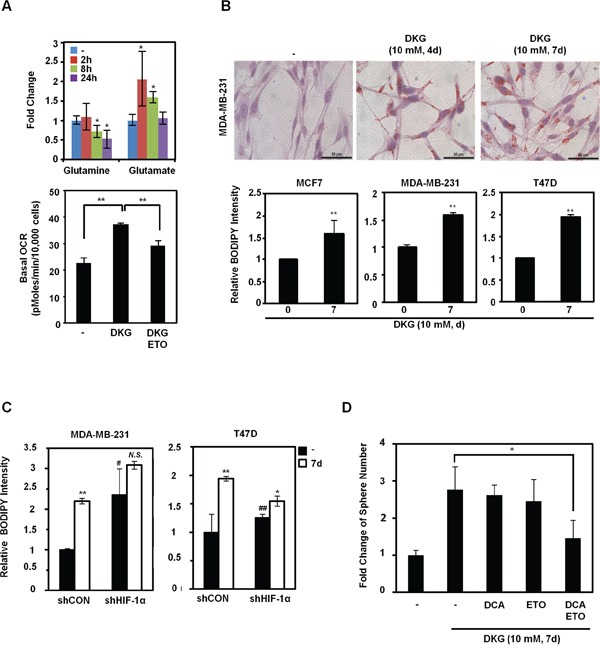
DKG regulates glutamine-dependent reductive carboxylation **A.** *Upper panel*: increased utilization of glutamine in DKG-treated cells. The levels of glutamine and glutamate in DKG-treated MDA-MB-231 cells were measured by NMR spectroscopy. _**_: *p* < 0.05; _**_: *p* < 0.01, n = 3. *Lower panel*: The enhanced OCR by DKG can be dampened by adding a fatty acid oxidation (FAO) inhibitor, etomoxir (ETO). OCR was measured 10 mins after ETO (40 nM) was added to MDA-MB-231 cells treated with DKG (10 mM, 4 days). n = 3. **B.** DKG promotes lipid droplet accumulation. *Upper panel*: Oil Red O staining was used to detect lipid droplets in DKG-treated MDA-MB-231 cells (10 mM, 4, 7 days). Bar: 50 μm. Representative images. n = 2. *Lower panel*: BODIPY 493/503 staining, followed by flow cytometric analysis, was performed to quantify the levels of neutral lipids in DKG-treated BC cells (10 mM, 7 days). _**_: *p* < 0.01, n = 3. **C.** The induction of lipid droplet accumulation by DKG is partially dependent on HIF-1α. BODIPY 493/503 staining was performed in DKG-treated BC cells harboring control shRNA or shRNA against HIF-1α. _*_: *p* < 0.05; _**_: *p* < 0.01; *N.S.*: not significant (DKG-treated *vs.* untreated). #: *p* < 0.05; ##: *p* < 0.01 (shCON *vs.* shHIF-1α), n = 3. **D.** Blocking both glycolysis and FAO reduces DKG-enhanced tumorsphere formation. MDA-MB-231 cells were pre-treated with DKG (10 mM, 7 days). Dichloroacetate (DCA) (10 mM) or ETO (10 μM) or DCA+ETO were added on day 5 and the cells were subjected to tumorsphere formation assays. _*_: *p* < 0.05.

From our NMR-based metabolite analysis, we also noticed that glutamine was decreased, whereas glutamate and citrate were increased, after DKG treatment (Figure 1C and 5A, *upper panel*). We postulated that, together with HIF-1α accumulation, the DKG-treated cells underwent reductive carboxylation, a process that utilizes glutamine to generate fatty acids. Indeed, the increased OCR in DKG-treated cells could be partially dampened by adding etomoxir (ETO), an inhibitor of carnitine palmitoyltransferase (CPT), which inhibits FAO [52] (Figure 5A, *lower panel*). We further speculated that lipids accumulated in DKG-treated cells. As expected, we detected elevated lipid droplet accumulation in DKG-treated MDA-MB-231 cells using Oil Red O staining (Figure 5B, *upper panel*) and a lipophilic fluorescent dye, BODIPY [53] (Figure 5B, *lower panel*). Notably, the magnitude of accumulated lipid droplets was partially dependent on the HIF-1α level. HIF-1α knockdown raised the basal lipid level and reduced the magnitude of induction (Figure 5C). Together with the results shown in Figure 5A, it is conceivable that DKG treatment also increases lipid utilization, presumably by FAO. To investigate a specific role of altered metabolism in the acquisition of tumorigenic properties, we pre-treated MDA-MB-231 cells with DKG for 4 days, added inhibitors of glycolysis (dichloroacetate, DCA) and FAO (ETO) for another 3 days, and assessed tumorsphere formation. As shown in Figure 5D, although treatment with DCA or ETO alone did not affect the tumorsphere number or other DKG-mediated phenotypes, including the CD133-positive or BODIPY-stained MDA-MB-231 fraction (Figure S4), combined DCA and ETO treatment notably reduced the tumorsphere number without affecting cell viability (data not shown).

To assess whether stabilization of HIF-1α alone was sufficient to mimic DKG and promote acquisition of tumorigenic phenotypes, dimethyloxaloylglycine (DMOG), an established PHD inhibitor, was used to stabilize HIF-1α. Unlike DKG, DMOG exclusively increased ECAR, at the expense of OCR (Figure S5A). Moreover, DMOG was less effective in enriching for the CD133-positive subpopulation (Figure S5B) and promoting lipid droplet accumulation (Figure S5C) than DKG, in MDA-MB-231 cells. However, DMOG and DKG induced HIF-1α and OCT4 abundance to a similar level in MDA-MB-231 and MCF7 cells (Figure S5D). Lastly, DKG rescued cells from death by long-term Glc deprivation (Figure S5E), but DMOG did not (Figure S5E), further distinguishing DKG from DMOG. Altogether, we concluded that the stabilization of HIF-1α is required, but alone is not sufficient, to establish the full suite of tumorigenic properties in BC cells. Therefore, we propose that increasing HIF-1α alone is not sufficient to promote tumorigenic phenotypes.

## DISCUSSION

**Figure 6:**
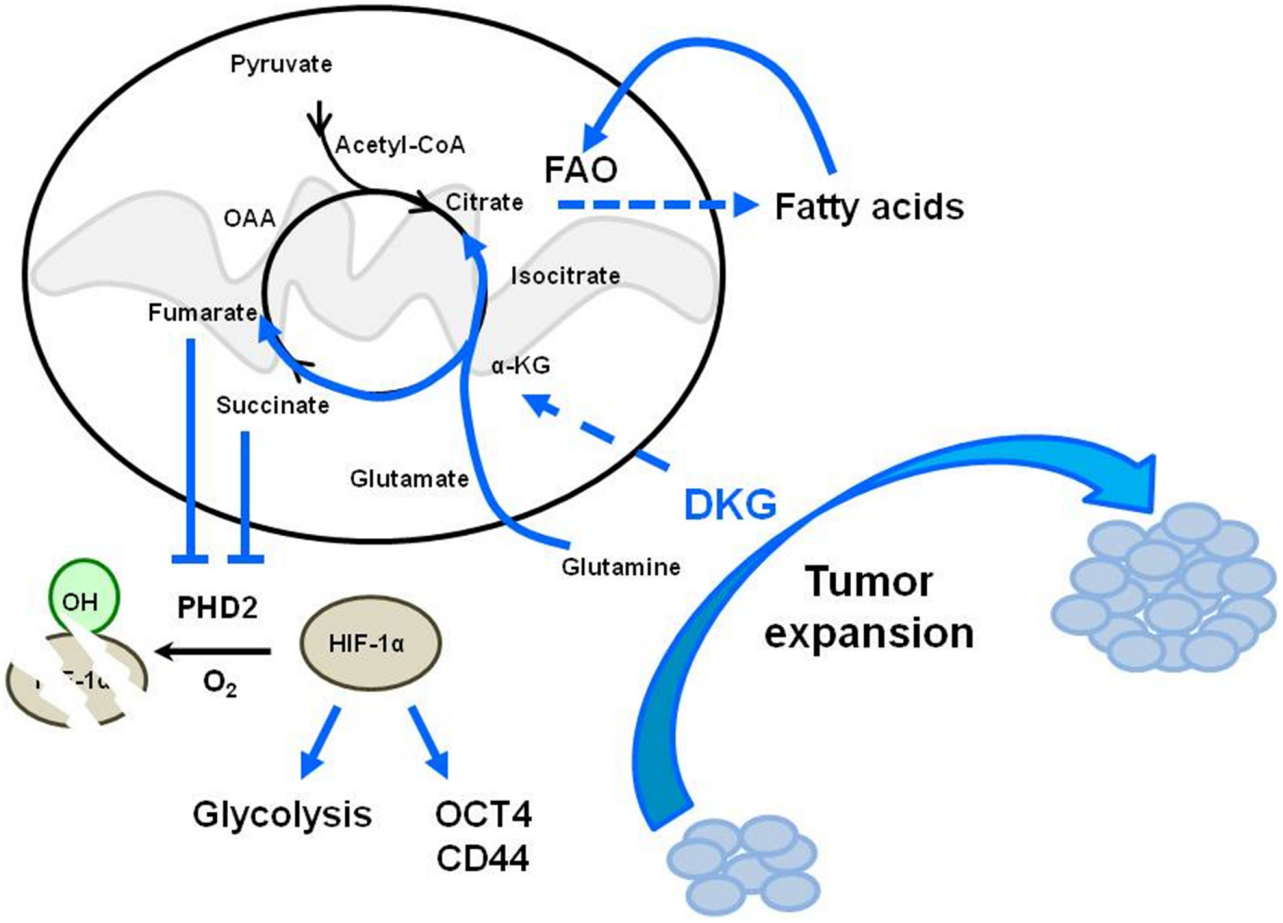
Model depicting the dual function of DKG to induce tumorigenic properties in BC cells through co-opting a pseudohypoxic pathway and metabolic rewiring. DKG promotes both OXPHOS and HIF-1α-dependent glycolysis to enhance tumorigenic expansion.

There is a growing appreciation for the idea that cancer cells undergo comprehensive metabolic reprogramming, leading to a glycolytic metabolic state that permits rapid growth and adaptation to the tumor microenvironment [1, 6, 54, 55]. Nonetheless, whereas altered glycolysis, glutaminolysis, and lipid synthesis have been demonstrated [6, 56], whether the exogenous nutrient availability alters the intrinsic tumor cell properties has not been clearly elucidated. Here, for the first time, we provide a comprehensive analysis of BC cell metabolic reprogramming, using DKG to uncouple mitochondrial respiration and also to increase succinate and fumarate. In response to DKG, the excess succinate/fumarate is then transported out of the mitochondria into the cytoplasm, where it impairs the activity of PHD, leading to the stabilization and activation of HIF-1α, and creating a state known as pseudohypoxia [32, 57]. This HIF-1α-induced pseudohypoxia then increases the glycolysis and oxygen flux rates. We propose that this HIF-1α-induced pseudohypoxia, together with increased FAO and transcriptional re-programming, promotes the acquisition of a tumorigenic CSC-like phenotype (Figure 6).

It is intriguing to note that the DKG-induced pseudohypoxic phenotype persisted for up to three passages (Figure 2A), which was further corroborated by an increased CAIX expression in tumors derived from DKG-pretreated BC cells (Figure 5D). Supported by the finding that CAIX is predominantly expressed in high-grade tumors [58], we propose that induction of a sustained pseudohypoxic state, with mitochondrial dysregulation, induces tumorigenicity.

The current studies reveal an important, but counterintuitive, ability of DKG to trigger a combined metabolic and transcriptional response, promoting a tumorigenic phenotype. DKG has been widely used as an analogue of α-KG, which is important for the TCA cycle, fatty acids and glutamine metabolism, and α-KG-dependent enzyme function. Therefore, one might suspect that an α-KG analogue would increase PHD enzyme function and de-stabilize HIF-1α. However, because DKG also increases the succinate and fumarate levels, the overall ratio of succinate/fumarate to α-KG remains high and PHD enzymes are inhibited, thereby increasing HIF-1α levels.

Although our *in vitro* and *in vivo* findings have established that increased HIF-1α levels, generated by DKG, are indispensable for the DKG-promoted tumor expansion, we cannot rule out the potential contribution of other α-KG-dependent enzymes. For instance, a group of jumonji domain-containing histone lysine demethylases (KDMs) utilizes α-KG as a cofactor for histone demethylation. α-KG is also important for DNA demethylation by the ten-eleven translocation (TET) enzymes [59]. The potential role of these and other DKG targets will require further investigation.

Our results are in marked contrast to a recent report indicating that a low intracellular ratio of succinate to α-KG is important to maintain the proliferation and the pluripotency of mouse embryonic stem cells in glutamine-free media containing inhibitors of GSK-3β and MAPK/ERK [34]. It has been shown that pluripotent stem cells have compromised mitochondrial function compared to differentiated cells [60]. Therefore, we speculate that there are important differences between the mouse ESCs and differentiated BC cells in intrinsic metabolic preferences and maintenance of the ratio of succinate/fumarate to α-KG. Also, there might be additional effects caused by the inhibition of GSK-3β and MAPK/ERK signaling pathways, as reported by Carey et al. [34], on the metabolic state. We therefore propose that increasing the ratio of succinate/fumarate to α-KG may result in opposite effects in ESCs and BC cells. Because both succinate and α-KG are crucial for regulating cellular signaling, the effective ratio of succinate to α-KG may be pre-determined by the cellular context and experimental paradigm.

It is well known that tumorigenic cancer cells or CSC-like cells increase tumor malignancy, but how cancer cells retain or acquire tumorigenic properties remains elusive. Metabolic reprogramming, such as the Warburg effect, is emerging as a mechanism for meeting the cancer cell's need for efficient anabolic metabolism. In the current study, we describe a model whereby cells acquire or maintain tumorigenicity by metabolic reprogramming, without additional mutations or exogenous manipulation of proto-oncogenes or tumor suppressor genes. The importance of tumor tissue niches, such as the hypoxic niche, in tumorigenicity, has been recognized [16, 61]. However, the impact of key nutrients on tumorigenic populations remains largely unknown. The classic CSC model follows a unidirectional, hierarchical structure, whereby CSCs differentiate into non-CSC progeny cells [11]. Recent studies have revealed that cues from the environment perturb the phenotypic equilibrium of cancer cell populations and enable the bidirectional inter-conversion of BC cells [12–14]. Based on our results, we further postulate that altered metabolism, induced by the nutritional microenvironment, could regulate cancer cell plasticity by promoting the conversion of non-CSC to CSC cells.

Moreover, the DKG-induced overall metabolic phenotype is consistent with previous reports on the role of HIF-1α in tumor expansion. It has been shown that CSCs, or tumorigenic cells, rely on glycolysis and using DCA to inhibit glycolysis can eliminate CSC populations and retard tumor growth [62]. Therefore, there is interest in the importance of mitochondrial metabolism in cancer progression and the maintenance of tumorigenicity [7, 8, 63]. Additional studies also highlighted a critical role for reductive carboxylation, mediated by hypoxia or HIF-1α, in promoting the lipogenic phenotype and supporting tumour growth [36, 56, 64–66]. Recently, a report indicated that ovarian cancer spheroid cells with tumorigenic properties demonstrate hypoxia-resistant metabolism, including enhanced glycolysis, pentose phosphate pathway activity and reductive carboxylation [67]. Another study demonstrated that leukemic cells exhibit increased FAO, uncoupled with ATP synthesis, and rely on *de novo* lipogenesis [52]. It is possible that DKG treatment of BC cells increases HIF-1α-dependent glycolysis and reductive carboxylation, which fuels FAO. Consistent with reports that FAO is a key mechanism of pancreatic tumor relapse [68] and is implicated in resistance to oxidative stress in glioblastoma cells [69], we demonstrate that inhibiting both glycolysis and FAO partially reduces DKG-induced tumorsphere formation.

In summary, metabolic reprogramming and the switch to glycolysis in cancer cells have received a substantial amount of interests over the past several decades, but the metabolic state has been poorly linked to the cancer cellular hierarchy. Our results suggest that breast cancer tumorigenicity can increase due to metabolic reprogramming and HIF-1α-dependent signaling. Treatment with the α-KG precursor, DKG, causes a cascade of metabolic changes and the induction of pluripotency markers, contributing to tumor expansion *in vivo*. Notably, cancer cells have multiple systems to sense energy imbalance and can modify signaling pathways to favor survival and growth. Along these lines, a dramatic increase in the prevalence of obesity and related metabolic disorders over the last few decades is associated with increased mortality from heart disease, stroke, diabetes, and cancer [70]. The nutrient excess associated with the obese state may affect cancer risk and aggressiveness in previously unknown ways, and this is an area of active research. Reminiscent of our report, the key TCA cycle intermediary metabolite, α-KG, is elevated in female *ob/ob* mice [71] and morbidly obese patients [72]. Our results are the first, to our knowledge, to provide insight into the potential tumor-promoting effects of excess exogenous α-KG. Therefore, our findings support a more comprehensive approach when devising therapeutic strategies in cancer, which should consider not only the presence of oncogenes and tumor suppressors, but also how the nutritional microenvironment might affect the subpopulation of cancer cells with tumorigenic CSC-like traits.

## MATERIALS AND METHODS

### Cell lines and reagents

All cell lines were cultured in a humidified 5% CO_2_ incubator at 37°C. The human BC cell lines MDA-MB-231, MCF7 and MDA-MB-468 were all obtained from American Type Culture Collection (ATCC) and cultured in Dulbecco's modified Eagle's medium (DMEM) containing 10% fetal bovine serum (FBS) and penicillin (100 U/ml)-streptomycin (100 μg/ml). T47D cells obtained from ATCC were cultured in RPMI supplemented with 10% FBS and antibiotics. MDA-MB-231 cells transduced with control shRNA (shCON) or shRNA targeting HIF-1α (shHIF-1α) were maintained in full DMEM containing puromycin (1 μg/ml). DKG, DCA, ETO and DMOG were purchased from Sigma-Aldrich.

### Human primary BC cells

Human primary BC cells, BC_01 and BC_03, were derived from patients treated at Taipei Medical University according to Institutional Review Board-approved protocols. Both BC_01 and BC_03 are invasive ductal carcinoma, ER-positive, PR-positive and HER2-negative. Isolated BC cells were grown on NIH J2 feeder cells in the presence of the Rho-associated protein kinase (ROCK1) inhibitor Y-27632 (Enzo Life Sciences). The growth medium contained 3:1 F12 nutrient mixture: DMEM with FBS (5%), EGF (10 ng/ml, Invitrogen), cholera toxin (10 ng/ml, Calbiochem), insulin (5 μg/ml, Gibco), Y-27632 (5 μM) and dexamethasone (0.1 μM, Sigma-Aldrich). The combination of the growth media and the usage of a J2 fibroblast feeder layer induces normal and tumor epithelial cells from many tissues to proliferate *in vitro* without transduction of exogenous viral or cellular genes [73, 74].

### Western blots and antibodies

For protein expression studies, whole cell lysates were extracted using SDS lysis buffer and then supplemented with cOmplete protease inhibitor mixture (Roche Applied Science). Equal amounts of whole cell lysates were separated by SDS-PAGE and immunoblotted with antibodies that recognized HIF-1α (BD Bioscience), OCT4 (BioLegend). Anti-GAPDH (Santa Cruz Biotechnology, Inc.) and anti-Actin (Millipore) antibodies were used to assess equal protein loading. Immunoblots were visualized using an enhanced chemiluminescence detection kit (ECL-Plus, Amersham Pharmacia Biotech) and were imaged with a Versadoc 3000 Imaging System (Bio-Rad). Densitometric data were obtained and quantified with Quantity One Software (Bio-Rad). Western analyses shown are representative of two to four independent experiments.

### NMR spectrometry-based metabolite measurement

The metabolites in the DKG-treated MDA-MB-231 cells were measured using solution nuclear magnetic resonance (NMR) spectroscopy. Briefly, metabolites from an equal number of cells treated with DKG (10 mM, 0, 2, 8, 24 h, three independent samples for each time point) were extracted as described previously [75]. The lyophilized, water-soluble metabolites were resuspended in 0.5 ml 99.96% D_2_O containing 50.9 μM 4,4-dimethyl-4-silapentane-1-sulfonic acid (DSS), which served as the ^1^H chemical-shift reference and an internal concentration standard. Three replicates were prepared. NMR spectra were acquired at 25°C on a Bruker Avance spectrometer equipped with a cryoprobe operating at 600 MHz ^1^H frequency. ^1^H NMR spectra were acquired with the Bruker noesygppr1d pulse sequence using a mixing time of 100 ms, recycle delay of 1 s and 2048 scans. Water suppression was achieved by saturation during the recycle delay and during the mixing time. The acquisition time is 4 s with 64 k data points and 13 ppm spectral width. 2D TOCSY spectra were acquired using the Bruker dipsi2phpr pulse sequence on one of each triplicate samples. The number of scans for each free-induction delay (FID) is 64. Complex data points of 4096 and 512 with spectra width of 13.3 and 11 ppm were used for direct and indirect dimensions, respectively. The recycle delay was 1.5 s, and the mixing time was 80 ms. The NMR spectra were processed using the Bruker topspin software or Chenomx, and the metabolite concentrations were calibrated against DSS using Chenomx NMR Suite Processor. The Chenomx NMR Suite Profiler software was used to identify the metabolites. 2D TOCSY spectra were used to confirm the assignments of some metabolites when ambiguity occurred in the 1D spectra. The fold changes were calculated by comparing the concentrations of metabolites (millimolar) in the treated samples (2, 8, 24 h) with the untreated samples (0 h).

### RNA sequencing and bioinformatic analysis

Total RNA was isolated from cells using the TRIzol reagent (Ambion) for RNA-sequencing (RNA-seq) according to the manufacturer's instructions. RNA-seq was conducted by the Integrative Genomics Core at City of Hope. Ingenuity Pathway Analysis was used for pathway analysis and functional annotation among untreated and DKG-treated cells. The genes involved in glycolysis and the electron transport chain were identified and then the heat map was generated using the GENE-E software (Broad Institute). For MDA-MB-231 cells, two biological replicates were subjected to RNA-seq analysis. The results were analyzed separately, as shown in the figures. For primary BC cells, one sample from each cell line was subjected to RNA-seq analysis. The RNA-seq data are available in Gene Expression Omnibus (GEO) database (GSE47031).

### Tumorsphere formation assay

The assay was performed according to a previous report [76]. The cells were seeded in triplicate in an ultra-low attachment plate (Corning) at 20,000 cells/ml. The spheres were counted 7 to 10 days after seeding the cells. Spheres with diameters ≥ 200 μm were enumerated and the bar graphs show the quantitative results from three independent wells. For serial passaging, day-3 tumorspheres were collected, dissociated by trypsin, and then seeded at 5,000 cells/ml and 1,000 cells/ml for the secondary and tertiary cultures, respectively.

### Immunofluorescent staining, intracellular ROS staining, flow cytometry and cell sorting

To detect cell surface markers, the cells were trypsinized, blocked in ice-cold PBS containing bovine serum albumin (BSA, 2%) and 0.1% NaN_3_, then incubated at 4°C with an anti-CD133/2 (293C3)-APC (Miltenyi Biotec), anti-CD44-APC or anti-CD24-PE (BD Pharmingen) antibody for 30 mins. ROS detection was done by DCFDA (Sigma) labeling followed by detection of the oxidized DCF signal. Cellular fluorescence was measured using 50,000 cells on an Accuri C6 Flow Cytometer (BD Biosciences). FACS was performed using an AriaSORT cell sorter (BD Biosciences). Data were processed using the FlowJo software package (Tree Star Inc.).

### ECAR and OCR

ECAR and OCR were measured as previously described [77] by the Seahorse Bioscience XF^e^24 Extracellular Flux Analyzer (Seahorse Bioscience) to track glycolysis and OXPHOS, respectively. The cells (6 × 10^4^) were seeded into each well the night before the measurements. The measurements were performed according to the manufacturer's instructions. Briefly, the cells were washed twice with XF Base medium (Seahorse Bioscience) plus L-Glutamine (2 mM final concentration), and the pH was adjusted to 7.35. ECAR was assayed by sequential injections of D-glucose (Glc; 10 mM), oligomycin (1 μM) and 2-deoxyglucose (2-DG, inhibitor of hexokinase; 50 mM; Sigma-Aldrich). OCR was also measured independently, as described previously [77]. The mito-stress test was performed by sequential injections of oligomycin (1 μM), FCCP (0.5 μM) and Rotenone (2.5 μM), according to the manufacturer's instructions. To integrate the ECAR and OCR data together, we chose the sixth point (22.5 mins after Glc injection) of the ECAR measurement for the ECAR vs. OCR plots. After the measurements, the cells were trypsinized for cell number normalization. The data are presented as the mean ± SD, n = 5.

### RNA extraction and qRT-PCR

Total RNA was extracted from cells using an RNeasy Mini Kit (Qiagen) according to the manufacturer instructions. The cDNA was synthesized from the total RNA using an iScript cDNA Synthesis Kit (Bio-Rad). Quantitative PCR analyses of targeted sequences were generated using the iTaq SYBR Green Supermix (Bio-Rad), a fraction of each cDNA sample, and gene-specific primers (Table S1). PCR amplification and fluorescence were detected using a MyIQ real-time PCR detection system, and threshold cycles were determined by the iCycler program (default setting). Fold induction was determined using the ΔΔC_T_ method, normalized to GAPDH.

### ATP assay

ATP production was measured using the ENLITEN^®^ ATP Assay System (Promega, FF2000) according to the manufacturer's instructions. The cells were harvested with cold PBS and then 5% trichloroacetic acid (TCA) was added. Tris-acetate-EDTA buffer (pH 7.75) was used to neutralize and dilute the TCA solution to a final concentration of 0.1%. The extracts were further diluted 1:100 and added to an equal volume of rL/L Reagent (Promega, FF2000). Then, the luminescence was measured using a TD-20e luminometer (Turner). The ATP standard (Promega, FF2000) was serially diluted to generate a regression curve for calculation of the exact number of ATP molecules, which was then divided by the cell number to derive the number of ATP molecules per cell.

### Lipid droplet staining

To visualize lipid droplets in the cells, Oil Red O staining was performed, as previously described [77]. BODIPY 493/503 (Life Technologies) staining was performed at 2.5 ng/100 μl and analyzed by an Accuri C6 Flow Cytometer (BD Biosciences).

### Tumor xenografts and isolation of tumor cells

To evaluate tumorigenesis, 10^4^, 10^3^, or 10^2^ untreated or DKG pre-treated cells were mixed with Matrigel (1:1, Corning) and injected subcutaneously in the left and right flanks of the same *NOD.Cg-Prkdc^scid^Il2rg^tm1Wjl^/SzJ* (NSG) mouse, respectively. Tumor weight was measured 2 months after injection. To further investigate the role of HIF-1α in the xenograft model, 10^4^ or 10^6^ of MDA-MB-231/shCON or shHIF-1α cells pre-treated with DKG were resuspended in 100 μl serum-free DMEM media, and then injected subcutaneously into the right flanks of six-week-old female NSG mice. The tumor volumes were measured twice weekly and the mice were euthanized on day 65, and the tumors were harvested and weighed. The tissue sectioning and the IHC staining were done by the Pathology Core at City of Hope, using an anti-CAIX antibody (Genetex), anti-CD44 antibody (Cell Signaling) and anti-CD133 antibody (Fitzgerald). Isolation and culture of tumor cells from the mouse xenografts were performed as previously described [78]. Briefly, the tumors were minced into small pieces and then digested in collagenase-hyaluronidase-DNase I (Sigma) buffer for 60 minutes at 37°C. The cell suspension was then passed through a 100 μm cell strainer (BD Biosciences) and the resulting cells were plated on petri dishes and maintained in the growth medium for culturing MDA-MB-231 cells.

### Ionizing radiation (IR)

A Shepherd Mark I Cesium-137 γ irradiator was used to irradiate cells at a fixed dose rate of 1 to 2 Gy/min. 1-4 Gy was used in this study.

### Clonogenic survival assay

DKG-pretreated cells were seeded in triplicate in six-well plates at 100 cells/well in complete growth medium one day before IR. After 10 days, cells were washed with PBS and fixed in ice-cold methanol for 10 mins, then stained with crystal violet solution (0.5%, 10 mins). Colonies were counted and the surviving fraction was calculated using the plating efficiency.

### Statistical analyses

Data are presented as the mean ± SD. Statistical significance was determined using the two-tailed Student's *t*-test. *p* < 0.05 is considered significant. _*_: *p* < 0.05, _**_: *p* < 0.01, _***_: *p* < 0.005.

## SUPPLEMENTARY FIGURES AND TABLES


