# Supplementary material for: HIF-1-alpha links mitochondrial perturbation to the dynamic acquisition of breast cancer tumorigenicity
Source: Oncotarget. 2016 Apr 4;7(23):34052–69. doi: 10.18632/oncotarget.8570 (PMC5085137; doi:10.18632/oncotarget.8570)
Supplement: Supplementary file 1 [file oncotarget-07-34052-s001.pdf]

HIF-1-alpha links mitochondrial perturbation to the dynamic acquisition of breast cancer tumorigenicity

SUPPLEMENTARY DATA

Flow cytometry for quiescent cells

To identify quiescent cell populations, the cells were trypsinized and then fixed in ice-cold 70% ethanol (v/v in PBS) prior to staining. The fixed cells were blocked, then incubated first with an anti-Ki67 antibody (Abcam), and second with an Alexa® Fluor 488 goat-anti-rabbit antibody (Invitrogen) and Hoechst 33342 (5 µg/ml, Sigma). The cellular fluorescence (FL1 for CD44 or Ki67; FL9 for Hoechst 33342) was measured using 50,000 cells on a Gallios Flow Cytometer (Beckman).

Flow cytometry for apoptotic cells

The apoptotic cells were stained using a FITC Annexin V apoptosis detection kit (BD Biosciences) according to the manufacturer's instructions. The cells were trypsinized, washed with PBS and resuspended in binding buffer prior to staining with Annexin V-FITC and PI. The apoptotic cells were detected by an Accuri C6 Flow Cytometer (BD Biosciences).

A

	α-KG (μM)	Succinate (μM)	Fumarate (μM)
-	19.8±4.8	10.8±3.0	2.2±0.3
2h	36.3±4.1	37.2±11.3	11.8±2.0
8h	36.3±0.6	41.3±3.2	13.2±1.2
24h	29.7±5.2	29.3±2.5	11.2±1.2

B

	Succinate/α-KG	Fumarate/α-KG
-	0.54	0.11
2h	1.02	0.32
8h	1.14	0.36
24h	0.99	0.38

C

Top Canonical Pathways

Name	p-value	Ratio
HIF1α Signaling	9.11E-05	28/103 (0.272)
Ubiquinol-10 Biosynthesis (Eukaryotic)	2.72E-04	7/12 (0.583)
Cell Cycle Control of Chromosomal Replication	3.1E-04	11/27 (0.407)
Antigen Presentation Pathway	5.03E-04	13/37 (0.351)
TGF-β Signaling	5.75E-04	23/87 (0.264)

D

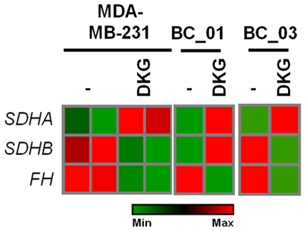

E

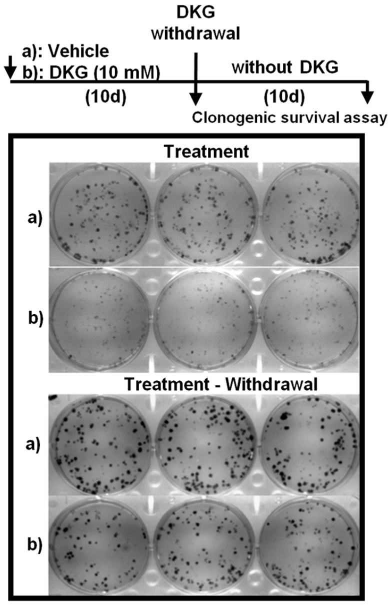

F

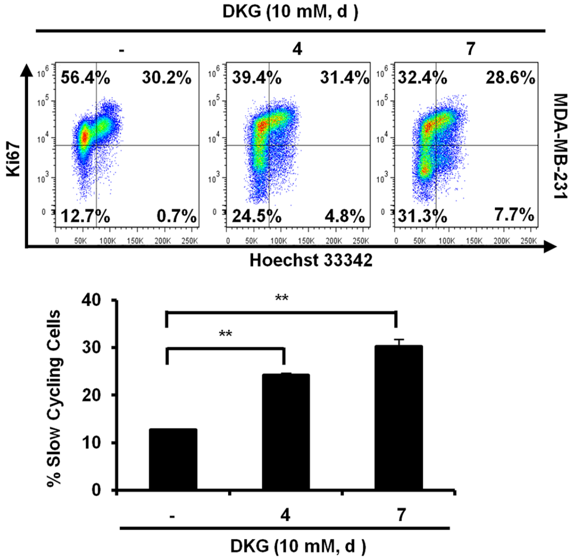

Supplementary Figure S1: DKG alters the ratios of TCA cycle metabolites and transcriptional landscape in BC cells. **A.** Table showing the intracellular concentration of α-KG, succinate and fumarate in MDA-MB-231 cells treated without and with DKG (10 mM, 2, 8, 24 h). **B.** Table showing the ratio of succinate to α-KG and fumarate to α-KG after the administration of DKG (10 mM, 2, 8, 24 h) in MDA-MB-231 cells. **C.** Top five pathways affected by DKG treatment from Ingenuity Pathway Analysis. **D.** *SDH* and *FH* mRNA abundances are decreased in DKG-treated BC cells. Heat map generated from the RNA-seq results showing the relative mRNA abundance of *SDH* and *FH* in DKG-treated MDA-MB-231 and two primary BC cells. **E.** DKG reversibly inhibited the clonogenicity of MDA-MB-231 cells. MDA-MB-231 cells were grown in colony-forming conditions and treated with DKG (10 mM) for 10-days (*top 2 panels*) or MDA-MB-231 cells were pre-treated with vehicle or DKG (10 mM) for 10 days then grown for 10 days without DKG (*lower 2 panels*). **F.** *Upper panel*: MDA-MB-231 cells treated with DKG (10 mM, 4, 7 days). A subpopulation of $Ki67^{Low}$ and Hoechst 33342^{Low} cells is shown in the G0 fraction; the numbers representing the percentage of G0 cells. *Lower panel:* quantitative results from the lower-left quadrants. **: $p < 0.01$, n = 3.

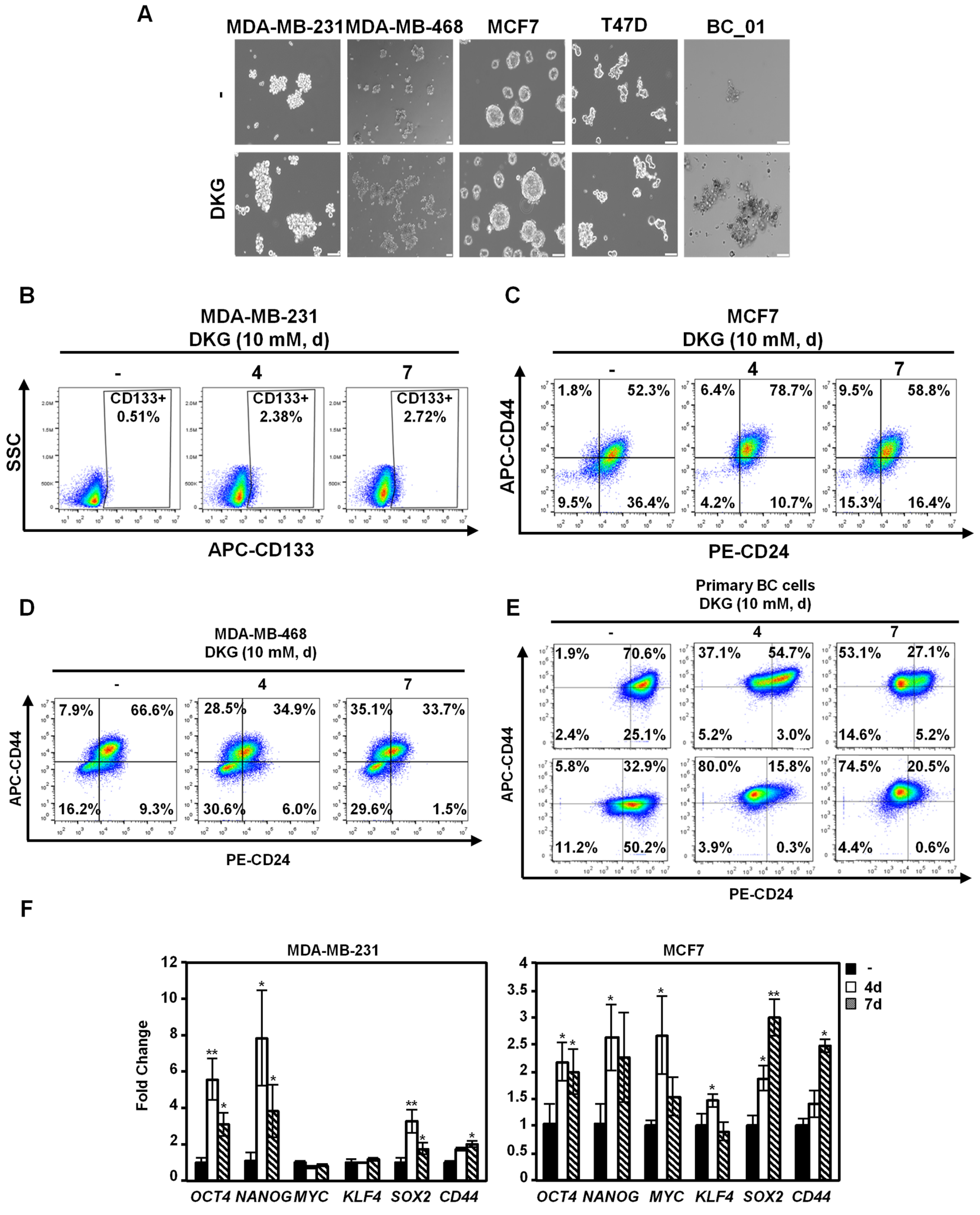

Supplementary Figure S2: DKG enriches the tumorigenic subpopulation in BC cells. A. Images showing mammospheres formed by the untreated and DKG pre-treated BC cells. Bar: 100 μm; 50 μm for MDA-MB-468 cells. **B-E.** Graphs showing flow cytometric analysis of surface markers in DKG-treated BC cells. APC: allophycocyanin-conjugated; SSC: side scatter. PE: phycoerythin-conjugated. **F.** DKG regulates the message abundance of pluripotency transcription factors. qRT-PCR was performed to assess the mRNA expression of stemness-associated transcription factors and CD44 in the untreated and DKG-treated BC cells (10 mM for 4, or 7 days). Fold induction was determined using the $\Delta\Delta C_T$ method normalized to *GAPDH*. $_*$: $p < 0.05$; $_{**}$: $p < 0.01$, n = 3 biological samples.

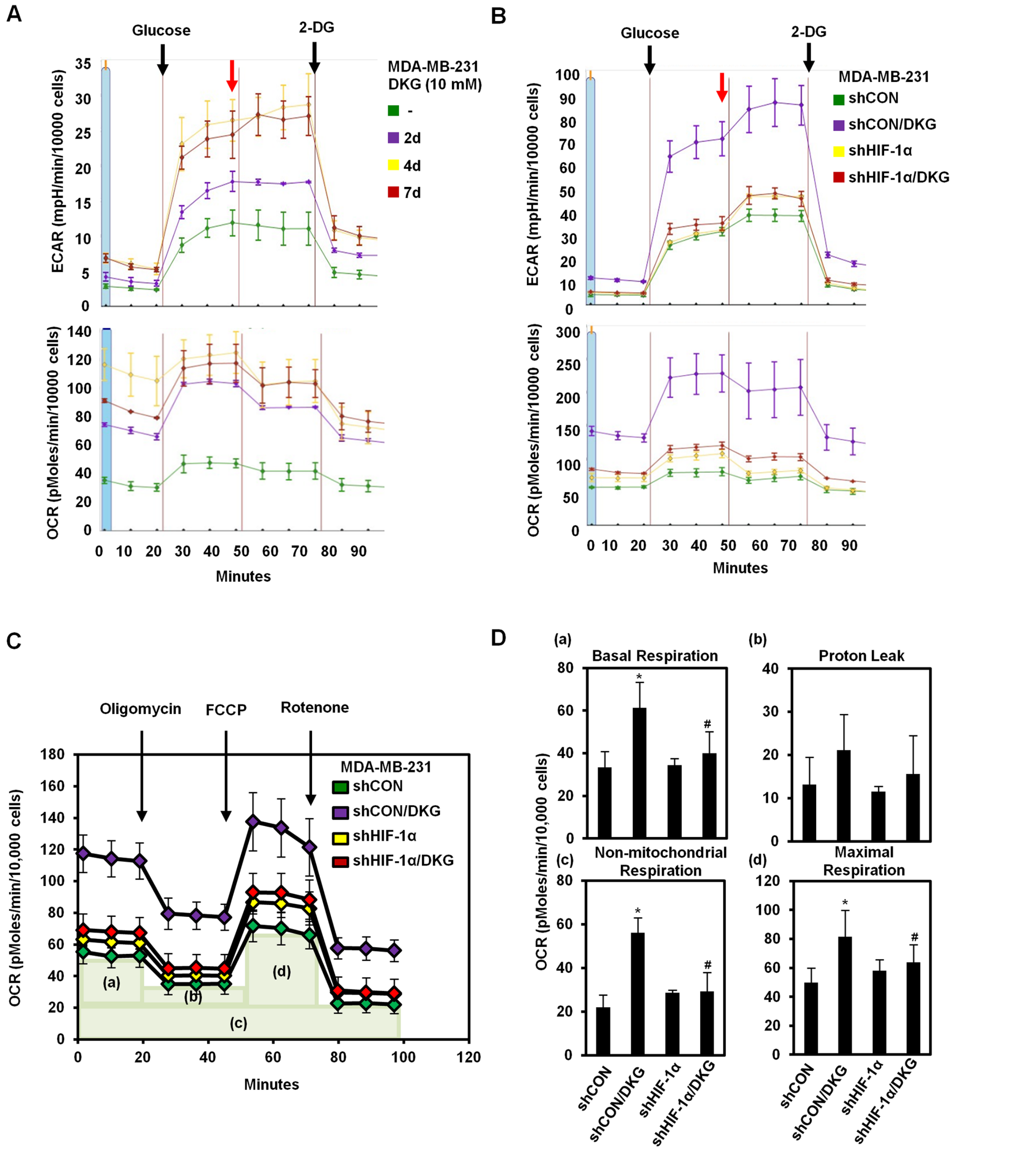

Supplementary Figure S3: DKG alters the metabolic landscape in BC cells. A. DKG increases OCR and ECAR. Glucose (Glc, 10 mM) and 2-DG (50 mM) were added at the indicated points. The red arrow represents the time point used for generating the ECAR versus OCR PhenoGram profile. The metabolic switch is shown in Figure 3A, *upper panel*; n = 3. *Upper panel*: ECAR; *lower panel*: OCR. 2-DG: 2-deoxyglucose. **B.** Knockdown HIF-1α impairs DKG-increased OCR and ECAR. Glc (10 mM) and 2-DG (50 mM) were added at the indicated points. The red arrow represents the time point used for generating the ECAR *vs.* OCR PhenoGram profile. The metabolic switch is shown in Figure 3A, *lower panel*; n = 3. *Upper panel*: ECAR; *lower panel*: OCR. **C.** DKG induces oligomycin-insensitive respiration. MDA-MB-231/shCON and MDA-MB-231/shHIF-1α cells treated with DKG were subjected to a mito-stress test to measure OCR. FCCP: Carbonyl cyanide-4(trifluoromethoxy)phenylhydrazone. **D.** Analyses of basal respiration (*panel a*), proton leakage (*panel b*), non-mitochondrial respiration (*panel c*) and maximal respiration (uncoupled respiration, *panel d*), as defined in (C), are shown. *: $p < 0.05$ (shCON *vs.* shCON/DKG); #: $p < 0.05$ (shCON/DKG *vs.* shHIF-1α/DKG).

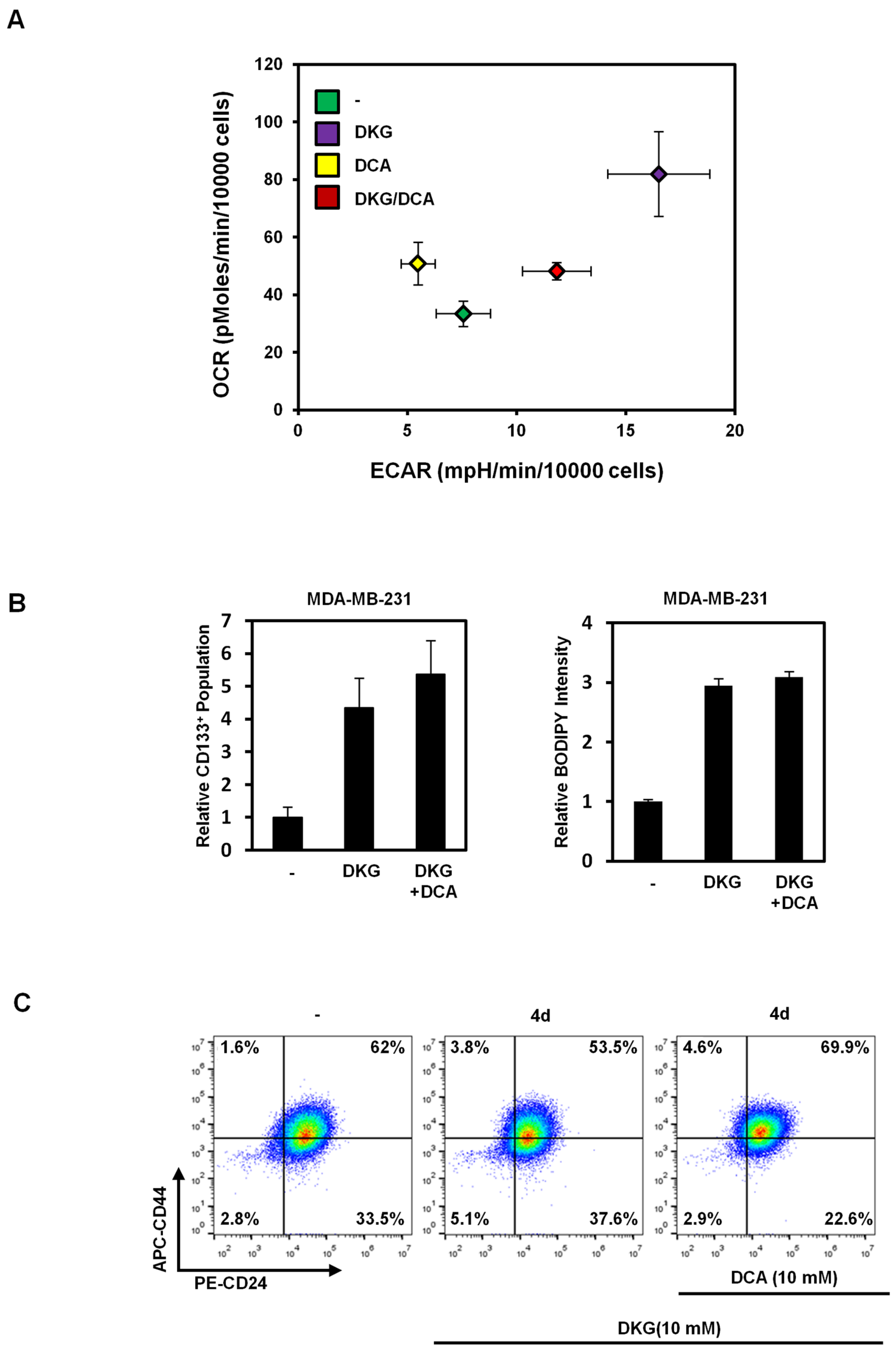

Supplementary Figure S4: DCA, a PDK inhibitor, does not affect the DKG-induced phenotypes. A. DCA reduced the ECAR and OCR induced by DKG. Measurement of energy metabolism was performed using the Seahorse extracellular flux analyzer. **B.** The CD133-positive population (*Left panel*) and lipid accumulation (*Right panel*) were quantified in the MDA-MB-231 cells treated with DKG (10 mM, 7 days) or co-treated with DKG and DCA (10 mM, 7 days). **C.** The CD44^{High}CD24^{Low} population was analyzed in the MCF7 cells after treatment with DKG (10 mM, 4 days) or co-treatment of DKG and DCA (10 mM, 4 days). $_{**}$: $p < 0.01$; *N.S.*: not significant.

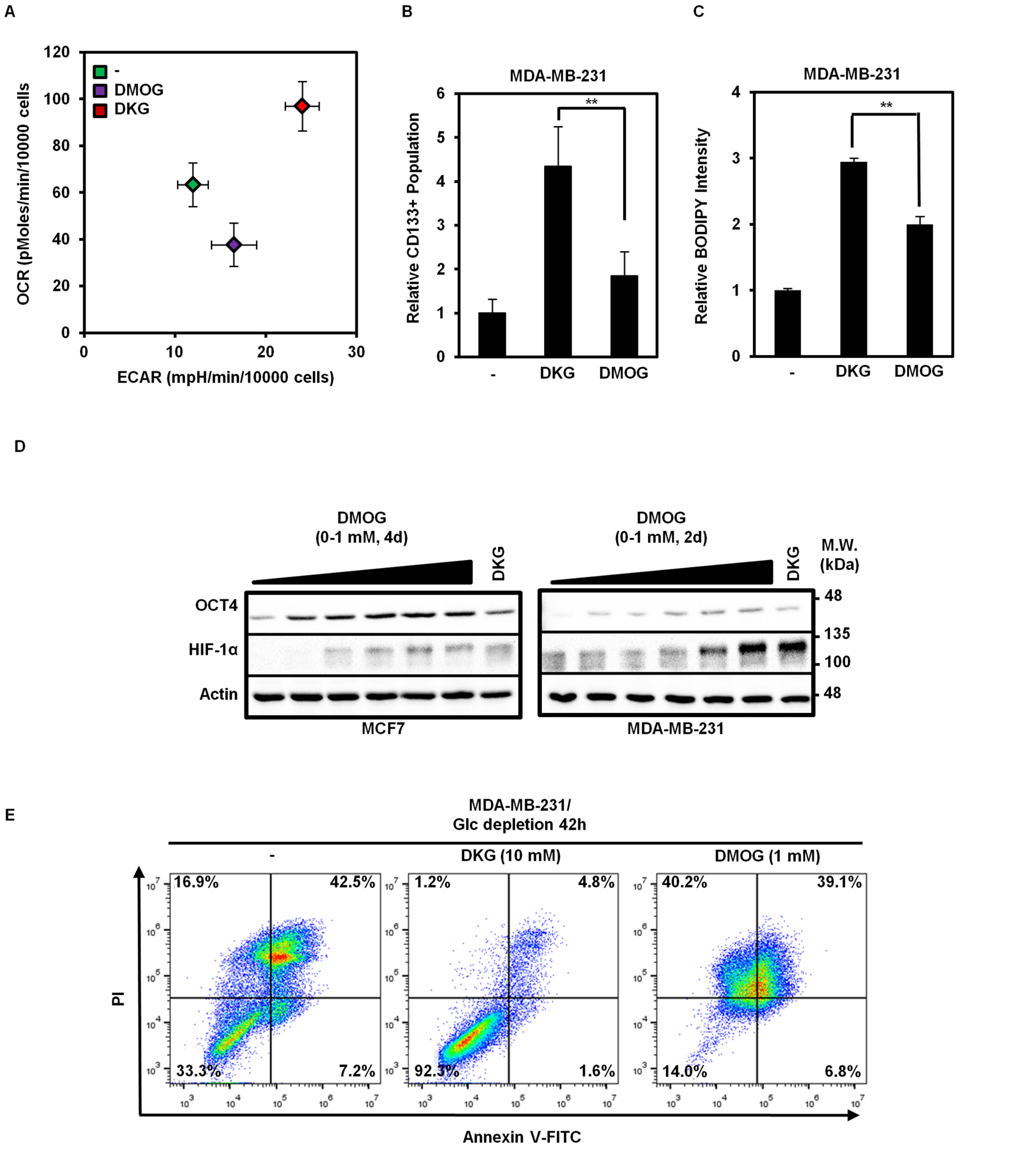

Supplementary Figure S5: DMOG-induced HIF-1α signaling does not fully recapitulate the effects of DKG on the BC cells. A. DMOG increased ECAR only, but reduced OCR. Energy metabolism in the MDA-MB-231 cells treated with DKG (10 mM, 4 days) or DMOG (1 mM, 4 days) was measured and analyzed using the Seahorse extracellular flux analyzer. **B, C.** The CD133-positive population (B) and lipid accumulation (C) were assessed in the MDA-MB-231 cells treated with DKG (10 mM, 7 days) or DMOG (1 mM, 7 days). **: $p < 0.01$, compared with the cells treated with DKG. n = 3. **D.** DMOG was also able to induce OCT4 expression in BC cells. The levels of OCT4 and HIF-1α were assessed in MCF7 or MDA-MB-231 cells exposed to either DMOG or DKG for the indicated times, by western blot analysis. Representative blots; n = 3. **E.** DKG, but not DMOG, was able to rescue the MDA-MB-231 cells from Glc starvation. Co-staining of Annexin V-FITC with propidium iodide (PI) was performed to detect apoptosis in the Glc-depleted cells treated with DKG or DMOG.

Supplementary Table S1. **List of primer pair sequences used in this study**

Gene	Forward primer sequence	Reverse primer sequence
OCT4	5'-CCTGAAGCAGAAGAGGATCACC-3'	5'-AAAGCGGCAGATGGTCGTTTGG-3'
NANOG	5'-CTCCAACATCCTGAACCTCAGC-3'	5'-CGTCACACCATTGCTATTCTTCG-3'
MYC	5'-CCTGGTGCTCCATGAGGAGAC-3'	5'-CAGACTCTGACCTTTTGCCAGG-3'
KLF4	5'-CATCTCAAGGCACACCTGCGAA-3'	5'-TCGGTCGCATTTTTGGCACTGG-3'
SOX2	5'-GCTACAGCATGATGCAGGACCA-3'	5'-TCTGCGAGCTGGTCATGGAGTT-3'
CD44	5'-CTGCCGCTTTGCAGGTGTA-3'	5'-CATTGTGGGCAAGGTGCTATT-3'
GLUT1	5'-ATTGGCTCCGGTATCGTCAAC-3'	5'-GCTCAGATAGGACATCCAGGGTA-3'
PDK1	5'-GAGAGCCACTATGGAACACCA-3'	5'-GGAGGTCTCAACACGAGGT-3'
GAPDH	5'-CCCCTTCATTGACCTCAACTA-3'	5'-CTCCTGGAAGATGGTGATGG-3'
